# Acquired Hemophilia A: Bleeding Pattern and Hemostatic Therapeutic Strategies

**DOI:** 10.3390/medicina59101739

**Published:** 2023-09-28

**Authors:** Renato Marino

**Affiliations:** Hemophilia and Thrombosis Centre, University Hospital of Bari, Piazza Giulio Cesare 11, 70124 Bari, Italy; remarino64@gmail.com

**Keywords:** anti-factor VIII inhibitor, bypassing drugs, recombinant porcine factor VIII

## Abstract

Acquired Hemophilia A (AHA) is a rare autoimmune disorder characterized by the onset of a sudden and unexpected bleeding episode in a patient with no personal or family history of bleeding diathesis, and with a typical laboratory feature, i.e., a prolonged activated partial thromboplastin time that is not otherwise explained. This bleeding disorder is caused by autoantibodies directed against the coagulation factor VIII (FVIII). AHA is idiopathic in 50% of cases and is secondary to well-defined diseases in the remaining 50%. AHA affects elderly patients although it has also been observed in the post-partum period. Bleeding manifestations are heterogeneous, ranging from mild to life-threatening bleeds involving limbs and organs. Severe bleeding with a significant decrease in hemoglobin levels must be promptly and adequately treated in order to avoid a worsening of the hemorrhages and their complications. According to international recommendations, the bypass agents (i.e., activated prothrombin complex concentrate and activated recombinant factor VII) and the replacement therapy with recombinant porcine FVIII are considered as the first-line therapy for bleeding control, due to their proven clinical efficacy. Plasma-derived or recombinant FVIII concentrates could be used as second-line treatments. Emicizumab may represent a valid and interesting therapeutic option for prophylaxis of bleeding recurrences.

## 1. Introduction

Acquired hemophilia A (AHA) is a rare bleeding disorder caused by the spontaneous development of neutralizing autoantibodies (inhibitors) directed against the endogenous coagulation factor VIII (FVIII). These autoantibodies inhibit FVIII activity, leading to a clinical picture similar to congenital hemophilia A [1]. The incidence of AHA in the general population is about 1.5 cases per million persons/years [2] and affects mainly the adult population, having rarely been described in children [3]. In two of the largest case series of AHA patients, the median age of subjects at diagnosis was 78 and 74 years, respectively, with more than two thirds of patients being older than 65 years [4,5]. Although AHA mainly affects elderly subjects, with no difference in incidence between males and females, several cases have also been reported in young women between 20 and 30 years in relation to pregnancy. About 50% of AHA cases are classified as idiopathic, since no underlying diseases have been identified. The remaining cases have been found to be related to autoimmune disorders (systemic lupus erythematosus, rheumatoid arthritis, thyroid disorders), hematologic or solid cancers, infections, dermatological diseases, drugs, and pregnancy [5]. Regarding pregnancy, AHA mainly occurs during the postpartum period, between one and four months, although cases have been documented up to one year after delivery. Anti-FVIII inhibitors have also been described during pregnancy or delivery, although less frequently; this setting is very challenging due to the high risk of severe uterine bleeding and hysterectomy [6].

### 1.1. Clinical Manifestations of AHA

The clinical definition of AHA is the sudden and unexpected onset of bleeding, spontaneously or after an invasive procedure, in a person with no personal or family history of hemorrhages. Diagnosis must be confirmed via laboratory investigation with the initial finding of a prolonged activated partial thromboplastin time (aPTT) that is not corrected after mixing equal volumes of both patient and normal plasma (mixing test). Considering that anti-FVIII inhibitor is typically time-and temperature-dependent, the absence of the correction of aPTT must be evidenced with a mixing test performed both at room temperature and after two hours of incubation at 37 °C. Once other causes responsible for aPTT prolongation have been excluded such as lupus anticoagulant, inherited coagulation factor deficiencies, heparin, or oral anticoagulant treatment, the diagnosis of AHA is confirmed through the evidence of low FVIII levels and finally via the detection of an anti-FVIII antibody, quantified in Bethesda Units (BU) [7].

The clinical picture of AHA is characterized by bleeding events of differing severities, from mild to life-threatening. One peculiar finding is that, unlike congenital hemophilia, the bleeding phenotype is not related to FVIII level or inhibitor titer detected at the time of diagnosis [8]. The most frequent bleeding manifestations of AHA are subcutaneous hemorrhages that are often very extensive, muscle hematomas everywhere in the body, mucosal bleeding (from the nose, gums, gastrointestinal and urogenital tracts), retroperitoneal bleeds and, prolonged bleeding after minor and major surgeries [4,5]. Bleeding into the muscles of the upper and lower extremities is very challenging because it may determine a compression on the adjacent neurovascular structures, causing a compartment syndrome with serious damage, often requiring urgent surgery [9]. Intracerebral hemorrhage is most often fatal, although fortunately uncommon. It is noteworthy that joint bleeds rarely occur in AHA, unlike with congenital hemophilia. In the European Acquired Hemophilia (EACH2) registry, 474 out of the 501 patients enrolled presented a bleeding at the diagnosis (94.6%). In more than two thirds of patients, the bleeds developed spontaneously, while in the remaining cases they were secondary to trauma, surgery, or partum. Additionally, in 70.3% of cases the bleeding event was classified as severe [10]. Interestingly, 315 out of 474 patients presented a single bleeding episode that was efficaciously resolved with hemostatic treatment, yet in the remaining 159 patients (33.5%), a bleeding recurrence was recorded (in the majority of the cases two bleeds) in 16 patients from four to seven episodes [10]. We have to consider that not all patients with AHA experience bleeding episodes at the diagnosis (30% of cases in the EACH2 registry) and cases where the hemorrhages are mild and insignificant. In these cases, a “wait and watch” strategy, without any hemostatic therapy, can be adopted [10]. However, it should be always kept in mind that, as long as the inhibitor is detected, even at a low titer, patients are at risk of developing hemorrhages at any time and in any part of the body with no criteria to predict the potential onset of major or even fatal bleeding episodes available [5]. For this reason, it is mandatory to promptly start a specific treatment in order to eradicate the anti-FVIII inhibitor in a short time [11]. In a recent study, Holstein et al. evaluated the bleeding risk and the efficacy of hemostatic therapy during 12 weeks of observation after starting immunosuppressive therapy (IST) in a cohort of 102 patients with AHA recruited in the prospective GTH-AH 01/2010 study [12]. In 80 patients, 148 bleeds were documented at presentation although, more importantly, 141 new bleeds occurred in 59% of the patients during the subsequent observation period at a mean rate of 0.27 bleeds per patient-week. Severe bleeds before starting IST or at presentation lasted a median of nine days compared with two days for those occurring in the follow-up period, emphasizing the importance of early diagnosis of AHA and prompt hemostatic treatment. The authors also demonstrated that weekly measured FVIII levels were significantly associated with the bleeding rate, yet the achievement of FVIII activity ≥ 50% eliminated the risk of bleeding, highlighting both the importance of starting the IST as soon as possible and the need of periodic monitoring of FVIII activity [12].

A particular critical issue is the onset of AHA in patients taking anticoagulant or antiplatelets drugs. In these cases, the diagnosis may be delayed since, in the first instance, the bleeding is attributed to these agents [13]. Therefore, excessive or recurrent bleeds in patients treated with antithrombotic medications should be further evaluated, especially in elderly adults who do not present an overdose of these drugs. It should be underlined that AHA diagnosis is always an indication for the discontinuation of anticoagulant and antiplatelet therapy [11].

In earlier studies, mortality related to bleeding was reported in 22–31% of the cases of AHA [14,15]. However, more recent studies have documented a lower frequency ranging from 3% to 9%*,* in particular, 3.2% in the EACH2 cohort [10] and 2.9% in the GTH-AH 01/2010 study [16], possibly due to a greater number of rapid diagnoses compared to previous years, along with the improved therapeutic approach.

### 1.2. Hemostatic Treatment of AHA

The therapeutic strategy of AHA is based on three cornerstones: arrest bleeding, start IST in order to eradicate the inhibitor and treat the underlying pathology (if identified).

The Italian Association of Hemophilia Centers emphasizes the concept that AHA patients must be managed in clinical centers where there is an expertise regarding the clinical and laboratory issues of this bleeding disease [17]. However, in reality, most subjects with suspected AHA are initially examined by physicians of several specialties with no clinical experience in treating patients with coagulation disorders (e.g., emergency room physicians, geriatricians, obstetricians, oncologists, rheumatologists, and surgeons) with limited knowledge regarding this disorder and its consequences if not promptly diagnosed and treated. For this reason, the implementation of clinical protocols shared between specialized centers is advisable, in order to provide the patient prompt and adequate support in the early stages of treatment, before being taken over by the reference center.

A rapid and appropriate management strategy is crucial, as the objectives are the control for acute bleeding and the concomitant treatment to eradicate the inhibitor, reducing the risk of the bleeds recurrence which persists if the anti-FVII inhibitor is present. Patients with severe bleeding and a significant decrease in hemoglobin levels require an immediate hemostatic intervention.

According to the updated international AHA treatment recommendation [11], the so-called bypass agents (BPAs), and the replacement therapy with recombinant porcine FVIII (rpFVIII) are indicated as first-line drugs for the treatment of clinically relevant bleeding in patients with AHA, and for prophylaxis in patients who are undergoing invasive procedures (GRADE 1B).

BPAs, namely recombinant activated factor VII (eptacog alfa) and activated prothrombin complex concentrates are commonly used for the bleeding treatment of patients with congenital hemophilia A and anti-FVIII inhibitor; they are able to overcome the interference of the inhibitor on the coagulation pathway, inducing thrombin generation regardless of the presence of FVIII [18].

Activated recombinant factor VII (rFVIIa) has demonstrated efficacy in bleeding control of patients with AHA. It is one of the most used drugs for this disease as evidenced in a Canadian AHA patients review and in the two large registries of Hemostasis and Thrombosis Research Society (HTRS) and EACH2, which documented its use in more than 50% of treated cases [10,19,20]. In the HTRS registry, bleeding resolution was reached in 85% of 139 reported episodes treated with rFVIIa [20], while the EACH2 registry evidenced a hemostatic efficacy of 91.8% of the bleeds treated with this drug [10]. Moreover, in a systematic review collecting 1714 bleeding episodes recorded in 1244 patients with AHA, a complete or partial hemostatic response was achieved in more than 90% of cases [21]. A large study carried out on 132 Japanese patients with AH showed a clinical response of 91% in 302 bleeding events treated with rFVIIa as a first-line treatment, using a median dose of 93.2 μg/kg [22]. Furthermore, a better hemostatic control was significantly reported in patients who received an initial dose of ≥90 μg/kg than patients treated with a dosage <90 μg/kg and in those who were promptly infused with the first rFVIIa after the onset of bleeding, highlighting the crucial importance of a rapid and adequate hemostatic intervention in these patients. rFVIIa, due to its short half-life, is administered intravenously at an initial dose of 90–120 μg/kg every 2–3 h and subsequently the drug infusion interval is gradually lengthened depending on bleeding severity, until its resolution (GRADE 1B) [10,16]. The limit of the frequent administrations of rFVIIa has been overcome by the demonstration of its stability for 24 h at 25 °C in a 50 mL polypropylene syringe, as reported in the Summary of Product Characteristics (SmPC). Therefore, using an infusion pump, the administration of rFVIIa is made simpler and more feasible, especially during intensive treatments [23].

The effectiveness of activated prothrombin complex concentrates (aPCC) is highlighted in several case reports and in a retrospective study of 34 patients in whom bleeding resolution occurred in 86% of cases [24]. In the EACH2 study (10), the efficacy of aPCC used as a first-line treatment was similar to rFVIIa (93%). In a recent Italian retrospective study conducted on 56 AHA patients by Zanon et al. aPCC, used as first-line therapy in 82% of cases at an average dose of 72.6 + 26.6 UI/kg, resolved 96.4% of bleeding episodes with a median of eight days of treatment. Furthermore, antifibrinolytic therapy was used in combination with aPCC in 40% of the treated bleeds and also in patients with cardiovascular morbidities. This combined treatment was efficacious, resulting in a shorter treatment duration and was well tolerated without thromboembolic complications [25]. Interestingly, aPCC has also been utilized as prophylaxis in patients with AHA and a high frequency of bleeding, resulting in a 50% reduction in bleeds; this regimen of short-term prophylaxis was performed after major bleeds for a mean period of 12.7 ± 5.7 days using a mean aPCC dose of 54 UI/Kg [26]. According to the guidelines, aPCC is administered through intravenous injection at a dose of 50–100 U/kg every 8–12 h, without exceeding a dose of 200 U/kg/day, until bleeding resolution (GRADE 1B) [11,17].

In the case of treatment failure with one first-line BPA, a switch to the alternative drug is indicated and should be performed quickly to avoid a worsening of the bleeding and a potential disabling sequelae (GRADE 1C) [17]. If rFVIIa and aPCC are not effective in bleeding control when used as single therapeutic agent, a concomitant use of both BPAs may be considered as reported in several case series of patients with congenital or acquired hemophilia A and anti-FVIII inhibitors. With this therapeutic approach, the BPAs were used following a combined or sequential protocol. In the first case, the drugs were administered simultaneously or one hour apart whereas in the second case, aPCC was infused every 6–12 h and 1–3 doses of rFVIIa were administered in the interval between the aPCC infusions. Although this type of treatment has proven effective in controlling previously unresponsive bleeds [27,28], it is burdened with an increased thromboembolic risk compared to the use of a single BPA, especially in elderly patients suffering from prothrombotic comorbidities. Ingerslev et al. in a retrospective analysis of nine patients with AHA treated with combined or sequential protocol of BPAs, recorded that one patient presented deep vein thrombosis and pulmonary embolism, one a fatal cerebral thrombosis, and three developed a disseminated intravascular coagulation. For this reason, the combined or sequential therapy should be used cautiously for treating bleeds that are unresponsive to the other available hemostatic drugs, and managed by physicians with expertise in treating patients with AHA (GRADE 2C) [29].

There is currently no standardized laboratory test available to monitor BPA and thus the efficacy of the treatment and the adjustments of dose and infusion frequency are based on both clinical criteria and the trend of hemoglobin levels. According to Tiede et al., the time to achieve an appropriate hemostasis should be between 6 and 24 h, depending on the site and severity of the bleeding. The resolution of bleeding and the absence of a bleeding relapse within two days of the end of the treatment is considered a feature of a complete hemostatic response. On the other hand, the lack of stability of hemoglobin levels, the need for blood transfusion, the increasing dimension of bleeds, and the persistence of pain are findings of a therapeutic failure [30].

Frequent rFVIIa or aPCC infusions increase the risk of thrombotic events. Fortunately, these events seem rare; in the EACH2 registry, 2.9% (5/174) and 4.8% (3/63) of patients with AHA who received rFVIIa or aPCC, respectively, presented thromboembolic complications [11]. Similar findings have been documented in other case series: for aPCC, 0–2.9% and for rFVIIa, 0–5% of treated bleeds [7].

Coppola et al. recommend BPAs as first-line hemostatic approach if the patient is hospitalized far from a specialized center and/or if the prompt and strict laboratory monitoring needed for the appropriate use of a replacement therapy is not guaranteed. These drugs are readily available and easily manageable even by physicians who are not an expert in hemorrhagic diseases, and in any case, under the supervision of specialized clinical centers.

Treatment with recombinant porcine FVIII (rpFVIII) concentrate (susoctocog alfa) was first described in a prospective clinical trial that enrolled 28 patients with AHA and a history of a major bleeding event. The initial dose administered was 200 IU/kg and both subsequent doses and the infusion frequency were decided by the investigator on the basis of a strict laboratory monitoring of FVIII levels, in order to maintain a FVIII activity of 80% in severe episodes, and 50% in the others bleeds. An effective or partially effective hemostatic response was observed in 100% of bleeds after 24 h and a complete bleeding control was achieved in 86% of patients [31]. The efficacy and safety of susoctocog alfa were confirmed more recently via small case series in which lower starting doses (100–120 IU/kg) were used [32,33]. Similar findings were also described in a recent Italian study where a mean starting dose of 100 IU/kg was administered to six out of nine patients, followed by mean doses of 50 IU/kg, every 8–12 h. After a median of four days of treatment, all the patients achieved a complete resolution of bleeding. Moreover, 66.7% of patients used rpFVIII as a second-line therapy and more than 70% of them had cardiovascular disease or thrombotic risk factors, without recording thromboembolic complications [34]. Although these interesting data regarding the efficacy of rpFVIII, also with a lower dosage than those used in the registrative trial are intriguing, the guidelines recommend starting treatment with susoctocog alfa with the approved dose of 200 IU/Kg (GRADE 1B) [11]. The great advantage of rpFVIII in comparison to rFVIIa and aPCC is the laboratory monitoring of plasma FVIII activity and thus the possibility of administering appropriate rpFVIII doses in order to avoid excessive FVIII levels which can be dangerous, especially to elderly patients with cardiovascular comorbidities. The main adverse event of this drug is the detection of anti-rpFVIII inhibitors, which can be present at baseline or can develop de novo during the rpFVIII treatment which is responsible, if present at high titers, for a treatment failure. A recent analysis of data from the prospective GTH-AH 01/2010 registry [35] showed that anti-rpFVIII antibodies were present in 44% of AHA cases at baseline and were associated with higher titers of antihuman FVIII inhibitor (>100 BU) and lower levels of FVIII (<3.9%). For this reason, it is advisable to check the anti-rpFVIII cross-reactivity before starting treatment and monitoring any development of anti-rpFVIII inhibitors during rpFVIII treatment. In particular, a close monitoring of the FVIII activity is required 30 min after each dose and before each subsequent dose, and anti-rpFVIII antibodies should be suspected if FVIII level peaks are significantly lower than expected [17]. To summarize, Coppola et al. recommended rpFVIII particularly for those patients with absent or low anti-rpFVIII cross-reactivity and in specific settings in which monitoring of hemostatic levels of FVIII can contribute to improving the efficacy and safety of the treatment, such as in the case of severe bleeding, surgery and/or in patients with high cardiovascular or thromboembolic risk. Moreover, rpFVIII should be considered for patients who are managed in specialized centers, where the presence of a laboratory available around the clock must be guaranteed. Finally, rpFVIII may represent a valid alternative after the failure of first-line treatment with BPAs [17].

Another therapeutic option for AHA is the replacement therapy with plasma-derived or recombinant human FVIII concentrates which are effective only in the presence of low inhibitor titer (<5 BU/mL). Moreover, their use is demanding as a large amount of the drug must be administered both to neutralize the inhibitor, and to ensure the presence of adequate circulating levels for the hemostasis [36]. Another drawback of this treatment is the potential risk of developing an anamnestic response, that is an increase in inhibitor titer after its administration, and for this reason a careful monitoring of FVIII:C levels and inhibitor titer is mandatory. In the EACH2 study [10], FVIII concentrates were successful in controlling bleeding only in 69.6% of cases compared with BPAs (93%). Tiede et al. suggested the use of human FVIII concentrate as a second-line therapy only when BPAs and rpFVIII are unavailable or ineffective and the inhibitor titer is low (GRADE 1B) [11].

Desmopressin is not considered as a therapeutic alternative considering its unpredictable hemostatic response and the potential adverse events due to fluid retention, especially in elderly patients (GRADE 1B) [11].

Table 1 summarizes the therapeutic agents available for bleeding management in patients with AHA.

### 1.3. Immunosuppressive Treatment

Once AHA is diagnosed, regardless of the presence of an active bleed, it is crucial to start an IST to allow a remission from the disease in a short time, considering the persistence of bleeding risk until the inhibitor is detected. According to Tiede et al., a complete remission is achieved with the restoration of FVIII levels within the normal values (>50%), the disappearance of anti-FVIII inhibitor and the absence of relapses after discontinuation of IST. The remission is considered partial when FVIII levels of 50% are reached and no bleeds occur 24 h after stopping hemostatic treatment [16].

The therapeutic agents mainly used are corticosteroids (prednisone or prednisolone), alone or in association with cyclophoshamide or rituximab. The EACH2 study documented a higher rate of complete remission in patients treated with the combination of corticosteroids and cyclophosphamide (77%), compared to patients treated both with corticosteroid alone and with rituximab in association with immunosuppressive agents (57% and 48%, respectively) [37]. Similarly, the GTH-AH 01/2010 study evidenced a complete remission only in 40% of patients treated with corticosteroids in monotherapy, unlike the patients treated with combined drugs (80%) [16]. This latter study also evaluated the possible presence of factors that could affect remission and survival in these patients. Interestingly, the authors reported that patients presenting a baseline FVIII level >1% and an inhibitor titer <20 UB reached more frequently a partial remission within 21 days of treatment when using corticosteroid as a monotherapy. On the other hand, patients with FVIII < 1% at presentation achieved less frequently and after a longer period the partial remission, yet also showed a lower rate of both complete remission and survival [16]. Based on these data, the recent international guideline on AHA suggests using, as first-line IST, corticosteroids alone in patients with a baseline FVIII > 1% e anti-FVIII inhibitor titer <20 UB. In patients with FVIII < 1%, it is suggested to start ITI on corticosteroids associated with cyclophosphamide or rituximab (GRADE 2B) [11]. The dosage recommended for corticosteroids is 1 mg/Kg/day per os for a period of 4–6 weeks, for rituximab it is 375 mg/Kg/m^2^ weekly for four cycles, and for cyclophosphamide it is 1.5–2.0 mg/day per os up to a maximum of six weeks (GRADE 2B) [11]. The international guidelines suggest, as alternative drug to cyclophosphamide, the administration of mycophenolate mofetil at a dosage of 1 g/day in the first week and subsequently 2 g/day (GRADE B) [11]. If the first IST fails with a specific protocol, the alternative drugs can be administered in a different combination as a second-line IST attempt. In several reports and case series, other therapeutic agents such as cyclosporine, tacrolimus, azathioprine, syrolimus and bortezomib were used to eradicate anti-FVIII autoantibodies as a second-line therapy, after the failure of the conventional drugs [38,39].

The GTH-AH 01/2010 study represents the first attempt to individualize the IST on the basis of well-determined prognostic markers (FVIII level, inhibitor concentration). Although these results need to be confirmed in larger studies, they constitute an important step towards standardizing the therapeutic strategy for inhibitor eradication. However, we must always keep in mind that we are facing elderly patients, in most cases with comorbidities. For this reason, a careful assessment and monitoring of the IST adopted is mandatory, considering the adverse events related to these drugs. Indeed, an incidence of 30% of treatment-related infections was described in these patients [40], representing an important cause of mortality in up to 47% of cases [16].

After the eradication of the anti-FVIII inhibitor through IST, relapses of AHA have been described. In patients enrolled in the EACH2 registry and treated with corticosteroids, corticosteroids plus cyclophoshamide and rituximab associated with other drugs, a relapse was documented in 18%, 12% and 4% of cases, respectively, after a median of 139 days of the inhibitor disappearance [37]. In a Dutch study, a relapse rate of 25% was reported after a median of 14.7 weeks from the discontinuation of IST [40]. For this reason, the guidelines recommend, after achieving a complete remission, carrying out a clinical and laboratory follow-up of the patients. Monitoring of the FVIII levels is suggested every month for the first six months and subsequently at progressively longer intervals, for a total period of at least two years (GRADE 1B) [11].

### 1.4. Future Therapeutic Approaches

Recently, in several case reports and case series, the off-label use of emicizumab in AHA patients was reported [41,42,43,44,45,46]. Emicizumab is a recombinant, humanized and bispecific monoclonal antibody with FVIII mimetic activity, approved for the prophylaxis of congenital hemophilia A with and without the anti-FVIII inhibitor [47]. A wide heterogeneity regarding the dosage and the infusion interval of emicizumab has been described in all the reported cases of AHA and the drug has been used both for the prophylaxis of bleeding and as treatment for acute bleeds (especially as a second-line therapy), resulting in efficacy control and the prevention of hemorrhages. An ongoing multicenter, single-arm phase 3 clinical trial (AGEHA) carried out in Japan will provide further important data about the efficacy and safety of emicizumab in patients with AHA [48]. The preliminary results documented 12 patients under IST, treated with emicizumab as a prophylaxis at 6.0 mg/kg on the first day, 3.0 mg/Kg on the second day and then at 1.5 mg/Kg/weekly from the eighth day onwards. During the treatment with emicizumab, five treated bleeds were recorded in two patients (16.7%) although without the features of a major bleed. Conversely, in the pre-treatment period, six patients (50%) had presented 30 bleeds, 27 of which were classified as major bleeding and treated with the usual hemostatic drugs. Only an asymptomatic deep venous thrombosis was detected during emicizumab treatment and was resolved without any treatment. It is noteworthy that the therapeutic protocol used for prophylaxis with emicizumab in AHA is different and more aggressive than the approved dosage for congenital hemophilia A, possibly with the aim of rapidly achieving a steady state of the drug in the circulation and thus an early clinical response [48]. Additional data on the AGEHA study have been published regarding patients with AHA who were initially considered ineligible for IST and started prophylaxis with emicizumab, which was effective and safe with no serious events (bleeding or thrombosis) reported [49]. Emicizumab could therefore represent an interesting alternative therapeutic option for AHA patients. Its potential main advantages are represented by the subcutaneous administration, faster resolution of bleeds, reduced requirement of traditional hemostatic drugs, reduced risk of rebleeding, earlier hospital discharge and outpatient follow-up, possibility of a less-intensive IST, and chance of postponement, especially in frail patients or those with contraindications to this therapy [50]. However, it must be taken into consideration that AHA affects elderly patients with prothrombotic comorbidities (cancer, cardiovascular disease) and women during pregnancy and puerperium, two conditions which increase the thrombotic risk. Pasca et al. in a recent review recorded, in 73 cases of AHA treated with emicizumab, three thrombotic episodes (two deep venous thrombosis and a minor stroke) and a patient who died during the treatment, albeit for reasons apparently unrelated to the drug [51].

## 2. Conclusions

AHA is a rare autoimmune disorder characterized by a heterogeneous clinical picture ranging from mild to severe or life-threatening bleeding. Current therapeutic agents, if administered early and at the appropriate dosages, are able to control the vast majority of bleeds, especially when considering the possibility of switching from one drug to another in the case of failed hemostatic response. Although a good safety profile has been described with the use of these drugs, the advanced age of AHA patients and the coexistence of comorbidities should be always taken into consideration in the proper choice and use of hemostatic agents. Starting as soon as possible, the IST is very important, as the bleeding risk persists until the anti-FVIII inhibitor is detected. Finally, in light of the increasing scientific data being published, emicizumab could represent a valid therapeutic alternative in the prophylaxis of bleeding for patients with AHA.

## Figures and Tables

**Table 1:** Therapeutic agents available for bleeding management of patients with acquired hemophilia A.

First-Line Therapy	Recommended Dosage	PROS and CONS
Bypassing agents		
Activated Prothrombin Complex Concentrate	50–100 IU/kg every 8–12 h until hemostasis is obtained, then at longer intervals as required Maximum dose: 200 IU/kg daily	PROS Proven efficacy for clinical bleeding Easily available Usable also with the absence of a specialized laboratory. CONS No specific laboratory assay to monitor efficacy and the appropriateness of dosages. Possible thrombotic risk
Activated recombinant Factor VII	90–120 μg/kg every 2–3 h until hemostasis is obtained, then at longer intervals as required	PROS Proven efficacy for clinical bleeding Easily available Usable also with the absence of a specialized laboratory. CONS No specific laboratory assay to monitor efficacy and appropriateness of dosages. Possible thrombotic risk
Replacement therapy		
Recombinant porcine Factor VIII	200 IU/Kg as starting dose, subsequent doses according to the clinical response, Factor VIII levels and the type or severity of bleeding; generally, infusions every 4–12 h	PROS Can be monitored with a simple assay. It determines a measurable increase in Factor VIII levels. Documented clinical efficacy (studies still limited) No thromboembolic complications described. CONS Less effective in cases of presence and/or development of anti-porcine Factor VIII antibodies Availability of a laboratory around the clock to monitor Factor VIII levels
Second-line therapy		
Plasma-derived or recombinant factor VIII	Variable depending on severity of bleeding, inhibitor titter, infusion modalities (bolus or continuous infusion)	PROS It determines a measurable increase in factor VIII levels. Proven efficacy in patients with low inhibitor titer (<5 UB) Easily available Can be monitored with a simple assay. CONS Possible anamnestic response High dose required. Strict laboratory monitoring (daily)

## Data Availability

Not applicable.
